# Oral–Systemic Health Burden and Dry Mouth as an Intermediary Factor: A Cross-Sectional Study in Singapore Nursing Homes

**DOI:** 10.1016/j.identj.2025.103934

**Published:** 2025-10-10

**Authors:** Sayaka Tada, Sarah Koh Mun Yee, Gabriel Lee Keng Yan, Tay Chong Meng, Sim Yu Fan, Clement Lai Wei Ming, Wong Mun Loke

**Affiliations:** Faculty of Dentistry, National University of Singapore, Singapore

**Keywords:** Nursing home, Dental health survey, Multimorbidity, Mouth dryness, Oral hygiene, Root caries

## Abstract

**Introduction and aims:**

This cross-sectional study aimed to assess the prevalence of oral and systemic health conditions among older adults in nursing homes (NHs) in Singapore and investigate associations between dry mouth and oral-systemic health factors.

**Methods:**

Participants were selected through a stratified cluster sampling method across 8 NHs. Inclusion criteria included age ≥60 years and physical/cognitive ability to undergo oral screening. Data on systemic health (care dependence, malnutrition, multimorbidity, and polypharmacy) and oral health (dry mouth, oral hygiene, dentition, gingival and periodontal condition, and denture quality) were collected in 2024. Bivariate analyses (Mann-Whitney U test and chi-square test) were used to assess associations between dry mouth and other health parameters. Logistic regression models were conducted to examine independent associations with dry mouth. Statistical significance was set at *P* < .05.

**Results:**

A total of 173 NH residents completed oral assessments (mean age: 75.0 ± 9.1 years; 38% female). Systemic health concerns were prevalent: 75.7% were care-dependent, 80.9% were malnourished or at risk, and 84.1% had multimorbidity. Cardio-cerebrovascular diseases (CVD) were the most common disease category (76.4%). Oral health problems were also widespread: 63.6% showed clinical symptoms of dry mouth, 46.6% had poor oral hygiene, 50.8% had at least one untreated coronal and/or root caries, and 89.2% showed gingival inflammation. Multivariate analysis identified CVD (OR = 3.99) as a systemic factor significantly associated with dry mouth. Greater plaque accumulation (OR = 5.09) and root caries (OR = 1.93) were also independently associated with dry mouth.

**Conclusions:**

This study provides the first comprehensive report on oral-systemic health burden in NH residents in Singapore. Dry mouth emerged as a potential intermediary factor linking systemic health conditions and dental deterioration. These findings underscore the importance of interprofessional care strategies to address dry mouth and its consequences in this vulnerable population. (292 words)

## Introduction

A high burden of oral health issues among older adults in residential long-term care (LTC) facilities, such as nursing homes (NHs), has emerged as a significant public health concern.^1^^,^^2^ Systematic reviews highlighted the high prevalence of poorly-controlled oral hygiene and dry mouth, untreated dental caries and periodontal disease, tooth loss, and denture-related complications is common across many developed nations.^3^^,^^4^ However, most studies originate from Europe, North America, and East Asia, with limited data from Southeast Asia. As aging accelerates in this region and increasing reliance on LTC, a clearer understanding of current oral healthcare needs is essential to guide effective interventions and eldercare policies.

Beyond regional gaps in epidemiological data, the link between systemic health and oral diseases remains insufficiently explored among older adults in NHs. Dry mouth may serve as an intermediary, commonly observed in care-dependent older adults due to factors such as xerostomic medications,^5^ polypharmacy,^6^ and underlying conditions, such as cardiometabolic disease.^7^^,^^8^ The natural decline in salivary function with age can further exacerbate oral dryness.^9^ In turn, dry mouth can result in a variety of dental complications,^10^ oral discomfort, difficulty swallowing and eating, and decreased taste sensation, all of which can adversely affect quality of life. Despite these clinical implications, the interplay between systemic health, dry mouth, and oral outcomes has been under-investigated in epidemiological studies, particularly in NHs. Moreover, many medical professionals view oral health as outside their scope of responsibility, leading to limited awareness or even scepticism about the clinical relevance of systemic–oral health interactions.^11^ A clearer understanding of this interrelationship may drive more integrated, interprofessional approaches to addressing the oral health needs of nursing home residents.

Singapore is one of the rapid aging nations in Southeast Asia,^12^ with the proportion of citizens aged ≥65 expected to reach 24.1% by 2030.^13^ While life expectancy has surpassed 83 years, the persistent 10-year gap with healthy life expectancy highlights increasing LTC needs.^14^ In response, the government has expanded LTC infrastructure,^15^, ^16^, ^17^ increasing NH capacity by 70% between 2010 and 2020, with plans to double it again by 2030.^18^ A wide range of eldercare services has also been introduced to ensure accessible and affordable solutions.^19^ However, despite these advancements, access to oral healthcare still seems limited in NHs due to the underdevelopment of domiciliary dental services.^15^ Although 2 small-scale studies in 1991^20^ and 2006^21^ illustrated a high prevalence of oral health issues among Singapore NH residents, no updated nationwide oral health survey has been conducted in nearly 2 decades.

Accordingly, this study aimed primarily to assess the prevalence of systemic and oral health status among older adults in NHs in Singapore and secondarily to explore the association of dry mouth with systemic and oral health factors.

## Material and methods

### Ethical considerations

This study focused on those physically and behaviourally capable to undergo oral health assessments, but it still involved those who were unable to provide independent consent. In such cases, a legally authorised representative provided consent on their behalf. If a participant showed unwillingness (eg, refused to open their mouth), the examination was discontinued to respect their autonomy. To minimise physical and psychological stress, oral assessments were conducted bedside or on wheelchair at their NHs by geriatric-trained dentists with limited to 10 minutes per session, with breaks provided as needed. We used only essential dental screening tools (mirror and tweezers), and therefore gingival and periodontal status were assessed via visual inspection rather than probing to minimise discomfort. Individual screening outcomes and treatment recommendations were shared with the NHs to support the follow-up. Written informed consent was obtained from subjects or legally authorised representatives. Ethical approval was obtained from the National University of Singapore Institutional Review Board (NUS-IRB-2021-909).

### Nursing home selection and recruitment

As of 2020, Singapore had 77 operational NHs: 12 large (>300 beds), 41 medium (150-300 beds), and 24 small (<150 beds). Given that Singapore is a compact city-state (approximately 750 km²), regional differences were minimal; Instead, variations were anticipated to be influenced by facility size, as larger NHs tend to have more structured resources and staffing. The initial recruitment goal was to include 13 NHs (2 large, 7 medium, 4 small), ensuring representation across facility sizes. Recruitment began at the community forum held in November 2019 organised by a local eldercare agency, where representatives from all NHs were invited to participate. Following the forum, 16 NHs agreed to participate in Phase 1 study (NH staff interviews). We recruited participants for this oral health survey from these NHs across 2021-2023. Due to recruitment and implementation challenges in the COVID-19 pandemic, the final sample consisted of 8 NHs: 2 large, 2 medium, and 4 small. Despite this discrepancy, the selected facilities spanned a broad range of sizes (45-450 beds), providing a meaningful cross-sectional analysis.

### Participant recruitment and sampling strategy

A stratified cluster sampling method was employed to select participants while ensuring representative coverage across NH sizes and adjusting for clustering effects within each NH. Of 1646 residents across 8 NHs, 644 met inclusion and exclusion criteria:

• Age: ≥60 years as of 2024.
• Physical Condition: Those with severe physical limitations or terminal illnesses (eg, excessive pain, respiratory distress) were excluded.
• Behavioural Condition: Those with aggressive behaviour due to advanced cognitive impairment or psychiatric disorders were excluded.

The minimum sample size to estimate the prevalence of systemic and oral health status was determined with a precision of 10%. Assuming a 50% prevalence rate for dental caries or gingival inflammation and a design effect of 2.0, accounting for the increased variability introduced by the cluster sampling design. To accommodate an expected 60% non-response rate, a total of 495 individuals were randomly selected (Table 1).

**Table 1:** Sample calculation.

NH	Size	Total residents	Eligible residents	Target sample size (B) (design effect = 2)	Random sampling size under expected 60% non-response rate	Agreed participants	Ratio of participants to eligible resident (%)
NH1	Large	450	101	31	78	27	26.7
NH2	Large	405	154	47	118	27	38.0
NH3	Medium	262	146	44	110	54	37.0
NH4	Medium	221	115	35	88	32	27.8
NH5	Small	105	86	26	65	31	36.0
NH6	Small	94	29	9	23	7	24.0
NH7	Small	64	5	2	5	3	60.0
NH8	Small	45	8	3	8	1	12.5
		**1,646**	**644**	**197**	**495**	**173**	**26.9**

Size: Large (>300 beds), Medium (150–300 beds), and Small (<150 beds).

### Data collection

Data collection was conducted between May and July 2024. A summary of the assessment protocol, variable definitions, and the validated measurement tools used is provided below; full details are reported in the Appendix.

• Systemic Health Information:
  i) Care dependence by the Katz Index^22^
  ii) Nutritional status by the Mini Nutritional Assessment – Short Form (MNA-SF)^23^
  iii) Medical diagnoses based on the latest available medical diagnostic records
  iv) Polypharmacy (the number of current medications)
• Oral Health Information:
  i) Dry mouth by the Clinical Oral Dryness Score^24^
  ii) Oral mucosal issues
  iii) Dentition (remaining teeth number)
  iv) Dental hygiene by Simplified Oral Hygiene Index^25^
  v) Dental caries
  vi) Gingival inflammation
  vii) Periodontal status (tooth mobility)
  viii) Denture quality
  ix) Denture hygiene
  x) Oral health-related quality of life by the OHIP-14^26^

Medical diagnosis and medication data were extracted from NH records, and the NH staff helped fill out the forms for care dependence and nutrition status. Visual Oral assessments using a dental mirror and tweezers were conducted by qualified dentists (ST, GL, TCM) with a paired assessment method, with 2 assessors working collaboratively during each session to cross-verify findings, enhancing reliability and minimising potential errors. All assessors were trained in advance to ensure consistency. The term “dry mouth” is used throughout this study to refer to observable signs of oral dryness.

### Statistical analysis

Descriptive statistics summarised participant characteristics. Bivariate analyses assessed associations between dry mouth and health parameters using Mann–Whitney U and Chi-square. Variables with *P* < .10 in bivariate analysis were entered into a multivariate logistic regression model, adjusted for age and gender, to identify independent associations with dry mouth. All statistical analyses were performed using IBM SPSS Statistics version 29.0 and statistical significance was defined as *P* < .05.

This study adhered to the STROBE guideline.

## Results

### Study participants

Of the randomly selected residents from the 8 NHs, 189 agreed to participate in the study (non-response/refusal rate: 62%). 173 completed the survey;14 declined oral assessment due to a lack of willingness, and 2 passed away prior to the participation. Participants distribution across 8 NHs is shown in Table 1. The mean age of participants was 75.0 (SD: 9.1) years, ranging from 59 to 99 years old with 38% female.

### Systemic health status

A total of 75.7% of the participants were classified as severely or moderately care-dependent. Malnutrition was observed in 24.3%, while 56.6% were at risk of malnutrition. 84.1% of participants were multimorbidity (≥2 chronic status) with a median of 3.1 (SD: 1.5) disease categories. Polypharmacy (≥5 medications) was observed in 92.4% of participants. The prevalence of disease categories is shown in Table 2. The most prevalent disease category was cardio-cerebrovascular disease (CVD) (76.4%, n = 130/170), including 40 participants with stroke, including both Cerebrovascular Accident and Transient Ischemic Attack. 29.4% were diagnosed with neurocognitive disorder, while 71.1% were not able to comprehend the questionnaire used in the survey such as OHIP-14 and/or provide meaningful responses.

**Table 2:** Prevalence of systemic health conditions.

Systemic health		n (%)
Care dependence	Independent	33 (19.1)
	Mild dependent	9 (5.2)
	Moderate dependent	24 (13.9)
	Severe	107 (61.8)
Nutritional status	Normal	33 (19.1)
	Malnutrition at risk	98 (56.6)
	Malnutrition	42 (24.3)
Polypharmacy	No	13 (7.6)
	Yes (5+ medication)	157 (92.4)
Multimorbidity	No	27 (15.9)
	Yes (2+ chronic conditions)	143 (84.1)
**Disease category**		**n (%)**
Cardio-cerebrovascular diseases		130 (76.4)
Psychiatric disorder		66 (38.8)
Diabetes mellitus		61 (35.9)
Neurocognitive disorder		50 (29.4)
Urologic disorder		50 (29.4)
Bone density disorder		29 (17.1)
Anaemia and other hematologic disorder		28 (16.5)
Joint disorder		24 (14.1)
Gastrointestinal disorder		22 (12.9)
Parkinsonism & other movement disorder		20 (11.8)
Chronic respiratory diseases		18 (10.6)
Hepatopancreatic disorder		18 (10.6)
Endocrine disorder		16 (9.4)

Note: Percentages are based on 173 participants unless otherwise indicated. Medical diagnoses and medication information from 3 participants were excluded due to outdated records (last updated over 2 years before the survey), resulting in a subsample of 170 for those variables.

### Oral health status

Oral health problems were highly prevalent (Table 3). Dry mouth was observed in 63.6%. 46.6% of dentate participants had poor dental hygiene, and 50.8% had at least one untreated coronal or root caries. The average number of remaining teeth was 7.65 (SD: 8.79), and only 13.9% had a functional dentition (>20 natural teeth). Although 61.8% being considered in need of denture treatment, over half did not use dentures. Among existing dentures 41.9% required repair or replacement. Gingival inflammation was present in 90% of dentate participants, and nearly a quarter had at least one tooth with moderate to severe mobility. Oral soft tissue lesions were uncommon. Among participants without a diagnosis of neurocognitive disorder (n=120), 50 participants were able to respond to the OHIP-14, the average score was 4.9 (SD: 7.47) with scores ranging from 0 to 33.

**Table 3:** Prevalence of oral health conditions.

Oral health		N (%)
Dry mouth (clinical oral dryness score, CODS)	Normal (CODS:0)	63 (36.4)
	Mild (CODS:1-3)	105 (60.7)
	Moderate (CODS:4-6)	4 (2.3)
	Severe (CPDS:7-10)	1 (0.6)
Tongue coat	None	31 (17.9)
	Mild	58 (33.5)
	Moderate	46 (26.6)
	Severe	38 (22.0)
Mucosal issues	Oral candidiasis	1 (0.6)
	Oral ulcer	2 (1.2)
	Denture stomatitis	7 (4.0)
	Angular stomatitis	2 (1.2)
Dentition	Edentulous (0)	53 (30.6)
	Partially dentate (1-9)	63 (36.4)
	Partially dentate (10-19)	33 (19.1)
	Partially dentate (20-27)	20 (11.6)
	Partially dentate (28 +)	4 (2.3)
Untreated retained root	Prevalence (≥1 Present) Average	62 (35.8) 1.57 (SD:3.03)
Dental plaque accumulation* (Plaque Index, PI)	Average PI	1.37 (SD: 0.81)
	Good (PI=0)	8 (6.7)
	Fair (0<PI<1)	56 (46.7)
	Poor (1<PI<2)	34 (28.3)
	Very poor (2<PI<3)	22 (18.3)
Dental calculus accumulation* (Calculus Index, CI)	Average CI	0.88 (SD: 0.85)
	Good (CI=0)	34 (28.3)
	Fair (0<CI<1)	56 (46.7)
	Poor (1<CI<2)	17 (14.2)
	Very poor (2<CI<3)	13 (10.8)
Untreated coronal caries*	Prevalence (≥1 Present) Average number	51 (42.5) 0.78 (SD:1.21)
Untreated root caries*	Prevalence (≥1 present) Average number	42 (35.0) 0.89 (SD:1.58)
Gingival inflammation* (most severe per person)	Healthy: No inflammation	13 (10.8)
	Mild: Swelling	94 (78.3)
	Moderate: Spontaneous bleeding	9 (7.5)
	Severe: Suppuration	4 (3.3)
Periodontal disease* (most severe per person)	Healthy: No mobility	55 (45.8)
	Mild: Less than 1mm	36 (30.0)
	Moderate: Between 1-2mm	19 (15.8)
	Severe: more than 2mm	10 (8.3)
OHIP-14 (n = 50)	Average Score	4.90 (SD: 7.47)
**Removable denture needs**		
Necessity of denture usage (n = 173)		107 (61.8)
Actual denture users among those who needs denture (n = 107)		40 (23.1)
Denture plaque accumulation†	Not visible	21(33.9)
	Less than 1/3	19 (30.6)
	Between 1/3 – 2/3	5 (8.0)
	More than 2/3	17 (27.4)
Denture quality†	Poor fitting	7 (11.3)
	Poor retention	22 (35.5)
	Poor stability	12 (19.4)
Necessity of denture repair/rebase†		14 (22.6)
Necessity of denture replacement†		12 (19.4)

Note: Percentages are based on 173 participants unless otherwise specified.

⁎ Plaque and periodontal assessments were limited to dentate participants (n = 120).

† Denture-related assessments were based on 62 existing dentures.

### Association between systemic health conditions and dry mouth (bivariate analysis)

Bivariate analysis (Table 4) showed care dependence, the number of disease categories, diabetes mellitus (DM), and CVD were significantly associated with dry mouth, while urologic disorders showed a borderline association. Other systemic conditions were not significantly associated.

**Table 4:** Association between dry mouth and systemic health conditions (bivariate analysis).

Systemic health	Dry mouth (Absence)	Dry mouth (Presence)	*P*-value
Age	74 (IQR: 69-80)	74.5 (IQR: 69-82)	.384
Gender			.384
-Male	42 (38.9)	66 (61.1)	
-Female	21 (32.3)	44 (67.7)	
Care dependence			.013*
-Independent & mild dependent	22 (52.4)	20 (47.6)	
-Moderate & severe dependent	41 (31.3)	90 (68.7)	
Polypharmacy (number of disease categories)	9 (IQR: 6-12)	10 (IQR: 7-13)	.149
Multimorbidity (number of disease categories)	3 (IQR: 1.75-4)	3 (IQR: 2-4)	.045*

**Each disease categories potentially relevant to dry mouth**			

Cardio-cerebrovascular diseases			.016*
-No	21 (52.5)	19 (47.5)	
-Yes	41 (31.5)	89 (68.5)	
Diabetes miletus			.016*
-No	47 (43.1)	62 (56.9)	
-Yes	15 (24.6)	46 (75.4)	
Neurocognitive disorder			.194
-No	48 (40.0)	72 (60.0)	
-Yes	14 (28.0)	36 (72.0)	
Psychiatric disorder			.199
-No	34 (32.7)	70 (67.3)	
-Yes	28 (42.4)	38 (57.6)	
Urologic disorder			.067
-No	49 (40.8)	71 (59.2)	
-Yes	13 (26.0)	37 (74.0)	
Parkinsonism & other movement disorder			.522
-No	56 (37.3)	94 (62.7)	
-Yes	6 (30.0)	14 (70.0)	
Chronic respiratory diseases			.418
-No	57 (37.5)	95 (62.5)	
-Yes	5 (27.8)	13 (72.2)	
Endocrine disorder			.619
-No	54 (35.1)	100 (64.9)	
-Yes	8 (50.0)	8 (50.0)	

Note: Values are presented as median (IQR: interquartile range) for continuous variables or n (%) for categorical variables. Statistical significance was determined using the Mann–Whitney U test for continuous variables, and Chi-square test for categorical variables. Variables with *P* < .05 are considered statistically significant.

⁎ *P* < .05.

### Association between dry mouth and dental conditions (bivariate analysis)

Participants with dry mouth had significantly higher dental plaque accumulation and the greater number of root caries. Coronal caries and gingival inflammation showed borderline associations, while tooth mobility was not associated (Table 5).

**Table 5:** Association between dry mouth and dental conditions (bivariate analysis).

Dental health	Dry mouth (Absence)	Dry mouth (Presence)	*P*-value
Dental plaque accumulation (Plaque index)	1 (IQR: 0.67-1)	1.27 (IQR: 1-2.04)	<.001⁎⁎
Coronal caries (Number of dental caries)	0 (IQR: 0-1)	0 (IQR: 0-1)	.076
Coronal caries (Prevalence: ≥1 present)	9 (30.0)	42 (46.7)	.110
Root caries (Number of root caries)	0 (IQR: 0-0)	0 (IQR: 0-2)	.047*
Root caries (Prevalence: ≥1 present)	6 (30.0)	36 (40.0)	.047*
Gingival inflammation			.064
-Healthy (no inflammation)	4 (35.7)	9 (64.3)	
-Mild (swelling only)	26 (28.4)	68 (71.6)	
-Moderate & severe (spontaneous bleeding + suppuration)	0 (0.0)	13 (100.0)	
Tooth mobility			.816
-No mobility	13 (23.6)	42 (76.4)	
-Less than 1 mm	11 (30.6)	25 (69.4)	
-1-2 mm	4 (21.1)	15 (78.9)	
-More than 2 mm	2 (20.0)	8 (80.0)	

Note: Values are presented as median (IQR: interquartile range] for continuous variables or n (%) for categorical variables. Statistical significance was determined using the Mann–Whitney U test for continuous variables, and Chi-square test for categorical variables. Variables with *P* < .05 are considered statistically significant.

⁎ *P* < .05;

⁎⁎ *P* < .01.

### Multivariate analysis of factors associated with dry mouth

A multivariate logistic regression assessed independent associations with dry mouth, including all variables with *P* < .10 in bivariate analysis. “Gingival Inflammation” was treated as a continuous variable reflecting increasing clinical severity. This approach was adopted to preserve the ordinal nature of the variable while avoiding instability from small subgroup sizes, particularly in the Moderate and Severe groups. After adjusting for age and gender, CVD remained significantly associated with dry mouth (OR = 3.99), while higher plaque accumulation (OR = 5.09) and the presence of root caries (OR = 1.93) were significantly associated with dry mouth (Table 6).

**Table 6:** Association between dry mouth and oral-systemic health conditions (multivariate analysis).

Potential factors	OR	95% CI for OR	*P*-value
Age	0.98	0.91-1.05	.521
Gender -Male (ref) -Female	2.10	0.64-7.00	.223
Care dependence -Independent & mild dependent (ref) -Moderate & severe dependent	1.63	0.42-6.29	.476
Multimorbidity (Number of disease categories)	1.00	0.66-1.51	.995
Cardio-cerebrovascular diseases -No (ref) -Yes	3.99	1.03-15.42	.045*
Diabetes miletus -No (ref) -Yes	1.68	0.51-5.57	.394
Urologic disorder -No (ref) -Yes	1.14	0.25-5.12	.866
Dental plaque accumulation (plaque index)	5.09	1.91-13.5	.001⁎⁎
Coronal caries (number of dental caries)	1.04	0.56-1.91	.909
Root caries (number of root caries)	1.93	1.08-3.44	.027*
Gingival inflammation (0 = Healthy, 1 = Mild, 2 = Moderate & Severe)	0.88	0.26-2.96	.835

Note: Logistic regression model was used to examine factors associated with clinically observed dry mouth. The model was adjusted for age and gender. Gingival Inflammation was treated as an ordinal variable to reflect increasing severity and to avoid unstable estimates due to small sample sizes in the Moderate and Severe categories. Variables with *P* < .05 are considered statistically significant. OR: Odds Ratios; CI: Confidence Intervals.

⁎ *P* < .05;

⁎⁎ *P* < .01.

An exploratory mediation analysis was considered to evaluate whether dry mouth mediated the relationship between CVD and dental outcomes. However, since no significant direct association was found between CVD and either plaque index or root caries, further mediation analysis was not pursued.

## Discussion

This cross-sectional study highlights the high burden of systemic and oral health issues among NH residents in Singapore, including care dependency, multimorbidity, and poor oral hygiene and untreated dental diseases. CVD was independently associated with dry mouth, while greater plaque accumulation and root caries were independently associated with dry mouth. To the best of our knowledge, this is the first comprehensive report on the oral–systemic health burden in an aged Southeast Asian nation, and the first study to explore dry mouth as a potential intermediary between systemic health status and dental disease in this vulnerable population.

### Systemic health burden

This study confirms a significant burden of systemic health conditions among NH residents in Singapore, consistent with global trends. The high level of care dependency may reflect Singapore’s ageing-in-place policy, which promotes home-based care until institutional care becomes necessary.^16^ The national guidelines state that NH admission is limited to individuals with physical or mental disabilities who require daily assistance and have exhausted other community-based care options (eg, home nursing or day care services).^19^ This criterion likely contributes to the high prevalence of multimorbidity and polypharmacy observed. The most prevalent conditions observed in this study align with previous reports from the US and China, where multimorbidity in LTC settings was dominated by cardiovascular, metabolic, endocrine, and neurological conditions.^27^^,^^28^ The prevalence of DM observed in this study is slightly higher than previous reports from the US and several European countries, where estimates range from 20% to 30% among NH residents.^29^ This discrepancy may reflect the well-documented rise in DM prevalence within Singapore,^30^ where the national health survey in 2023 indicates a progressive increase in DM prevalence with age, reaching 22.3% among adults aged 70 to 74 years among community dwelling individuals.^31^ Given that our study population comprises vulnerable institutionalised people with a mean age of 75.0 years, this higher prevalence is likely by age-related risk accumulation and the increased likelihood of diabetes-related complications necessitating institutional care.

### Oral health burden

Oral health issues were highly prevalent in this study, consistent with global reports on institutionalised older adults.^3^^,^^4^ Dry mouth was observed in 63.6% of participants, though generally not severe, which is comparable to or exceeding prior studies.^6^^,^^32^ Poor dental and denture hygiene were common, despite denture care being a technically manageable task for caregivers. Furthermore, untreated dental caries, gingival and periodontal issues remained a significant concern. Despite the high prevalence of edentulism and the need for denture treatment, over half of those requiring dentures were not using them, and 41.9% of existing dentures required repair or replacement due to poor condition. These patterns mirror earlier findings from qualitative studies with dentists and physicians^11^^,^^33^ and suggest that the situation has not markedly improved since earlier local surveys in 1991 and 2006.^20^^,^^21^

In Singapore, oral health management, including hygiene assistance, assessment of dental needs, and referrals, is mandated under the NH regulations^34^ and is therefore likely being implemented in practice. However, the high prevalence of oral health problems observed in this study suggests that these efforts may not be yielding outcomes aligned with dental professional expectations. This may be partly attributed to typical staff make-up in NHs (ie, nurses/healthcare assistants and medical doctors) who are not specifically trained in oral healthcare to carry out these responsibilities. Without dedicated dental personnel in NHs, addressing oral health needs may remain a challenge,^35^^,^^36^ Additionally, several other barriers have been previously identified, including residents’ physical and cognitive limitations, logistical and financial constraints, and competing caregiving priorities.^35^ Addressing these challenges will likewise require coordinated, interprofessional efforts across all relevant disciplines.

### Link between systemic health conditions and dental problems

Dry mouth may serve as a key factor linking systemic health conditions and dental problems. In the bivariate analysis, care-dependency, multimorbidity, and CVD and DM were associated with dry mouth. In the final multivariate model, only CVD remained significantly associated, suggesting a more robust link with dry mouth. Although other factors lost statistical significance, its consistent bivariate association and known biological mechanisms still warrant clinical attention. These findings align with previous research indicating that metabolic and vascular health contribute to salivary gland hypofunction through both direct pathophysiological mechanisms^37^ and medication-induced xerostomia.^6^ CVD itself may not directly cause dry mouth, but commonly prescribed medications—such as β-blockers, diuretics, calcium channel blockers, ACE inhibitors, anticoagulants, and statins—are well-documented causes of reduced salivary flow.^7^ Likewise, dry mouth is a frequent symptom among patients with DM, possibly linked to hyperglycaemia-induced dehydration and medication.^38^ Interestingly, despite the plausibility of polypharmacy contributing to dry mouth,^39^ polypharmacy was not significantly associated with dry mouth in our study. This may be due to differences in assessment tools: while most studies examine xerostomia (subjective sensation), we used CODS, which captures only observable signs. Many participants of this study were unable to self-report, and CODS may have missed subtler, subjective experiences of dryness. This could explain the lack of a significant association in this study.

Among oral conditions, plaque accumulation and root caries were independently associated with dry mouth. Persistent salivary hypofunction promotes biofilm accumulation and caries through reduced buffering, diminished antimicrobial action, and impaired remineralisation.^40^^,^^41^ Among older adults, root caries is particularly common due to: i) greater exposure of root surfaces in older adults due to gingival recession,^42^ ii) lower mineral content of root cementum surfaces and dentin, which makes it more prone to demineralisation,^43^ and iii) reduced buffering capacity and antimicrobial action of saliva.^44^

This cross-sectional study cannot establish causality, and our exploratory mediation analysis did not demonstrate a statistically significant mediating effect of dry mouth between CVD and oral health outcomes. However, the systemic influences on salivary function and dental disease are multifactorial and cumulative in nature, which may not be fully captured by conventional statistical mediation models. Nevertheless, the observed pattern of associations remains consistent with dry mouth’s potential intermediary role in the oral–systemic health continuum. Given the high prevalence of CVD in this setting, training NH staff to detect and manage dry mouth may help mitigate its adverse effects.

### Limitations and strengths

A major limitation of this study lies in the recruitment challenges encountered during participant selection, particularly the underrepresentation of medium-sized NHs. As NH participation was voluntary, facilities with greater awareness or interest in oral health may have been more likely to participate. The 62% non-response/refusal rate may further contribute to bias, although such rates are common in NH-based studies. While these factors may affect generalisability, the use of stratified random sampling across different facility sizes still enhances the representativeness of the sample and provides a reasonably diverse cross-sectional snapshot. Importantly, the final sample of 173 participants yielded meaningful insights into the oral–systemic health status of NH residents. The data most likely reflect those who are more cooperative with oral healthcare interventions.

The non-invasive oral assessment, while ethically appropriate, may have led to an underestimation of the severity and accuracy of certain oral health conditions, particularly periodontal disease. However, this “senior-friendly” approach allowed for the inclusion of residents who may have lacked mental capacity but were still able to tolerate the oral assessment. This enabled the study to gather data from a broader range of NH residents, even those who might otherwise have been excluded due to more severe cognitive issues. In addition, geriatric-trained dental professionals conducted oral assessments with a paired assessment method to enhance inter-examiner reliability, ensuring consistent data collection. We were also able to collect systemic health information by extracting medical diagnoses and medication records directly from NH records, reducing reliance on self-reported information.

For this vulnerable population, using self-reported assessment tools such as OHIP-14 was challenging due to cognitive limitations. Assessors often felt uncertain whether participants fully comprehended the questions and provided meaningful responses. In fact, despite their poor oral health, the average OHIP-14 score was unexpectedly low, raising concerns about the tool's applicability in this setting.

### Future research direction

Conducting epidemiological studies in NHs is inherently challenging due to a combination of operational, ethical, and familial barriers.^1^ In our experience, institutional concerns about increased workload, difficulties in obtaining informed consent from family members, and the need to adhere to strict ethical safeguards significantly impacted the recruitment process. These barriers are commonly reported in similar research and have likely contributed to the overall scarcity of oral health data in this population. To overcome these challenges, we found that early engagement with NH leaders and collaboration with local eldercare agencies were essential. However, to scale such research and ensure broader institutional participation, formal government support or endorsement will be critical. Such backing can not only encourage trust and participation but also streamline research coordination across NH facilities.

Our experience also highlighted the high value of using simplified, minimally invasive oral assessment protocols which we tailored to the NH context. While these methods may sacrifice some diagnostic precision, they allowed for the inclusion of residents with cognitive impairments who were frequently excluded from research due to ethical or practical constraints. This approach holds promise for broader international application.

Future research may also benefit from incorporating simple yet objective oral function tests for higher-functioning residents to complement clinical findings. Lastly, a critical area for future investigation is the development of valid approaches to assess OHRQoL among individuals who are unable to verbally express their experiences. Capturing the perspectives of the most vulnerable should remain a central goal in both research and policy design for oral healthcare in long-term care settings.

## Conclusion

This study highlights a substantial burden of both systemic and oral health issues among nursing home residents in Singapore, with dry mouth serving as a potential intermediary linking systemic conditions, particularly CVD, to dental health deterioration. Addressing dry mouth could serve as a preventive strategy to mitigate oral health decline in this vulnerable population. While limitations in sampling and assessment tools exist, the findings underscore the need for stronger collaboration between dental, medical, and nursing care providers to ensure integrated and person-centred eldercare.

## Funding

This work was supported by the NUS (National University of Singapore) start-up grant (A-0002946-00-00).

## Data statement

Due to data protection, data sharing is only feasible upon reasonable request and in collaboration with the authors.

## Author contributions

**Sayaka Tada:** Conceptualisation, Methodology, Funding acquisition, Investigation, Data curation, Formal analysis, Writing – original draft, Writing – review and editing, Supervision. **Sarah Koh:** Investigation, Data curation, Project administration, Writing – review and editing. **Gabriel Lee Keng Yan:** Investigation, Writing – review and editing. **Tay Chong Meng:** Investigation, Writing – review and editing. **Sim Yu Fan:** Methodology, Formal analysis, Writing – review and editing. **Clement Lai Wei Ming:** Formal analysis, Writing – review and editing. **Wong Mun Loke:** Methodology, Writing – review and editing. All authors gave their final approval and agreed to be accountable for all aspects of the work.

## Conflicts of interest

All authors declared there is no conflicts of interest.

## Appendix Supplementary materials

Image, application 1

## Footnotes

Supplementary material associated with this article can be found in the online version at doi:10.1016/j.identj.2025.103934.
