# Supplementary material for: Oral–Systemic Health Burden and Dry Mouth as an Intermediary Factor: A Cross-Sectional Study in Singapore Nursing Homes
Source: Int Dent J. 2025 Oct 10;75(6):103934. doi: 10.1016/j.identj.2025.103934 (PMC12547251; doi:10.1016/j.identj.2025.103934)
Supplement: Supplementary file 1 [file mmc1.docx]

**Appendix Table. Systemic and Oral Health Examination Criteria**

| **Systemic Health Status** | |
| --- | --- |
| Care Dependence | Care dependence was assessed by the Katz Index(1) of Independence in Activities of Daily Living (ADL). This standardized tool evaluates six basic ADL domains: bathing, dressing, toileting, transferring, continence, and feeding. Each domain is scored as either dependent (0) or independent (1), resulting in a total score ranging from 0 to 6. Scores are categorized as follows: 0–1 (severe dependence), 2–3 (moderate dependence), 4–5 (mild dependence), and 6 (complete independence). |
| Nutritional Status | The Mini-Nutritional Assessment Short Form (MNA-SF)(2), a standardised tool for evaluating nutritional status in older adults, was used. The MNA-SF includes six questions and yields a total score of 14, with scores categorized as follows: 0–7 (malnutrition), 8–11 (at risk of malnutrition), and 12–14 (normal nutritional status). |
| Medical Diagnosis | The presence of 13 disease categories was determined based on the latest medical diagnostic records (2023-2024), according to the International Classification of Diseases (ICD):  1. Neurocognitive Disorder: Status marked by significant cognitive decline, affecting memory, attention, language, and problem-solving abilities. Examples include Alzheimer's disease and vascular dementia.  2. Psychiatric Disorder: Mental health status impacting mood, behaviour, and thought processes. This category encompasses depression, anxiety disorders, bipolar disorder, schizophrenia, and intellectual disabilities.  3. Cardio-Cerebrovascular Diseases: Status affecting the heart, blood vessels, and brain, such as coronary artery disease, arrhythmias, peripheral artery disease, and cerebrovascular diseases such as stroke and transient ischemic attacks.  4. Diabetes Mellitus: A metabolic disorder characterized by elevated blood glucose levels due to the body's inability to produce sufficient insulin or effectively utilize the insulin it produces.  5. Chronic Respiratory Diseases: Long-term status affecting the lungs and airways, including chronic obstructive pulmonary disease (COPD), pulmonary fibrosis, asthma, and bronchiectasis.  6. Hepatopancreatic Disorder: Status affecting the liver, pancreas, and biliary system. Examples include hepatitis, cirrhosis, and pancreatitis.  7. Gastrointestinal Disorder: Status affecting the digestive system> Examples include gastritis, gastroesophageal reflux disease, irritable bowel syndrome, haemorrhoids, constipation, and gastrointestinal bleeding.  8. Urologic Disorder: Disorders of the urinary tract and bladder, including neurogenic bladder, benign prostatic hyperplasia, urinary tract infections, and kidney stones.  9. Endocrine Disorder: Status involving hormonal imbalances due to dysfunction in the endocrine glands, such as hyperthyroidism, hypothyroidism, hyperparathyroidism, and parathyroid adenoma.  10. Anaemia and Other Hematologic Disorder: Status that affect the blood, including red blood cells, white blood cells, platelets, and clotting factors. Examples include anaemia, thrombocytopenia and cytopenia.  11. Parkinsonism & Other Movement Disorder: Neurological status that impair motor function, leading to symptoms such as tremors, rigidity, and bradykinesia. Examples include Parkinson's disease, essential tremor, cerebral palsy, hereditary spastic paraplegia, and multiple sclerosis.  12. Joint Disorder: Status that impair joint function, causing pain, stiffness, or inflammation. Examples include gout, osteoarthritis, rheumatoid arthritis, and rheumatism.  13. Bone Density Disorder: Status characterized by a decrease in bone mass and density, increasing the risk of fractures. Examples include osteoporosis and osteopenia. |
| Polypharmacy | The number of current prescribed medications was recorded. |
| **Oral Health Status** | |
| Dry Mouth | Dry mouth was assessed using the Challacombe Scale(3), or known as a clinical oral dryness score (CODS) to evaluate the severity of oral dryness based on clinical signs. The scale provides an additive score ranging from 1 to 10, with 1 indicating the least severity and 10 indicating the most severe dryness. Scores are categorized as follows: 1–3 (mild dryness), 4–6 (moderate dryness), and 7–10 (severe dryness). |
| Oral Mucosal Issues | The following mucosal issues were visually assessed.   - Denture Stomatitis: Redness and swelling of the mucosa beneath the denture. - Ulcer (Non-Denture Related): A round or oval sore with a yellowish or white base and a red halo. - Angular Stomatitis: Cracking, redness, and scaling at the corners of the mouth. - Candidiasis: White patches that can be wiped off to reveal a red base, red and flat lesions, or a combination of red and white lesions, affecting the tongue, soft palate, and oropharynx. - Abscess: A localised swelling filled with pus. |
| Oral Hygiene | Oral hygiene was visually assessed based on the extent of tongue coating across the entire tongue and classified as follows: No Coating (0% of the surface), Mild Coating (less than 1/3 of the surface covered), Moderate Coating (between 1/3 and 2/3 of the surface covered), and Severe Coating (more than 2/3 of the surface covered). |
| Dentition | The number of remaining natural teeth with coronal structure and retained root was recorded. |
| Dental Hygiene | Dental hygiene was visually assessed using the Plaque Index (PI) and Calculus Index (CI)(4), which score visible plaque or calculus on tooth surfaces. Scores ranged from 0 (no plaque/calculus) to 3 (extensive plaque/calculus covering the tooth surface), with severity increasing as plaque or calculus spread from the gingival margin to a larger portion of the tooth surface. The dental arches were divided into six sextants, and the highest score in each sextant was recorded. |
| Dental Caries | Dental caries was visually assessed by the following criteria: coronal caries (criteria: the presence of enamel breakdown) and root caries (criteria: the presence of softness and cavitation). |
| Gingival Inflammation | Gingival inflammation status was visually assessed with the category of (Swelling, Spontaneous bleeding, and pus leakage. Swelling means generally gingivitis or early periodontitis. |
| Periodontal Disease | Tooth mobility was assessed as an indicator of periodontal disease, using a tweezer with gentle force. The mobility was classified based on the degree of movement: less than 1mm (no significant mobility), 1mm to 2mm (moderate mobility), and more than 2mm (severe mobility). |
| Denture Quality | Denture quality was assessed by qualified dentists according to the following criteria.  [Fitting]   - Good: No visible gaps between the denture and oral tissues. - Acceptable: Minor discrepancies in the denture base adaptation, with minor adjustments needed. - Poor: Visible gaps between the denture base and the oral mucosa.   [Retention]   - Good: Denture resists dislodgement under strong pull during testing. - Acceptable: Denture holds but shows slight movement under moderate force during the pull test. - Poor: Denture easily dislodges with minimal force during the pull test.   [Stability]   - Good: Denture remains stable and shows no movement under lateral or anteroposterior testing. - Acceptable: Slight movement or rocking under lateral or anteroposterior pressure, but stable during normal function. - Poor: Denture rocks or shifts significantly when lateral or anteroposterior forces are applied. |
| Necessity of Denture Usage | The necessity for denture usage was assessed using the following criteria to identify individuals who benefit or may benefit from oral rehabilitation with denture. The criteria used to screen participants included:   - Lack of Functional Dentition: Participants lacking minimally functional dentition (defined as functional occlusion from the area of premolars to anterior teeth on both sides). - Communication Ability: The ability to understand and follow basic instructions provided by the dentist during the examination. - Oral Feeding Capability: The ability to eat orally, excluding individuals reliant on non-oral feeding methods such as tube feeding. - Cooperation During Examination: the capability to cooperate during the dental examination process. |
| Denture Hygiene | Denture hygiene was visually assessed on the mucosal surface of each denture and classified as follows: Clean (nonvisible plaque), Moderately Poor (plaque covering less than 1/3 of the surface), Poor (plaque covering less than 2/3 of the surface), and Very Poor (plaque covering more than 2/3 of the surface). |
| Oral Health Related Quality of Life | The OHIP-14 (Oral Health Impact Profile - 14)(5) was used. The total score is the sum of the scores for all 14 items, with possible scores ranging from 0 to 56. A higher score indicates a greater negative impact on the individual's quality of life due to oral health problems, while a lower score suggests a lesser impact. |

[References]

1. Katz S, Ford AB, Moskowitz RW, Jackson BA, Jaffe MW. Studies of Illness in the Aged: The Index of ADL: A Standardized Measure of Biological and Psychosocial Function. JAMA. 1963;185(12):914-9.

2. Rubenstein LZ, Harker JO, Salvà A, Guigoz Y, Vellas B. Screening for undernutrition in geriatric practice: developing the short-form mini-nutritional assessment (MNA-SF). J Gerontol A Biol Sci Med Sci. 2001;56(6):M366-72.

3. Osailan SM, Pramanik R, Shirlaw P, Proctor GB, Challacombe SJ. Clinical assessment of oral dryness: development of a scoring system related to salivary flow and mucosal wetness. Oral Surg Oral Med Oral Pathol Oral Radiol. 2012;114(5):597-603.

4. Greene JC, Vermillion JR. THE SIMPLIFIED ORAL HYGIENE INDEX. J Am Dent Assoc. 1964;68:7-13.

5. Slade GD, Spencer AJ. Development and evaluation of the Oral Health Impact Profile. Community Dent Health. 1994;11(1):3-11.
